# Food Metabolites as Tools for Authentication, Processing, and Nutritive Value Assessment

**DOI:** 10.3390/foods10092213

**Published:** 2021-09-17

**Authors:** Mariana C. Pedrosa, Laíres Lima, Sandrina Heleno, Márcio Carocho, Isabel C. F. R. Ferreira, Lillian Barros

**Affiliations:** Centro de Investigação de Montanha (CIMO), Instituto Politécnico de Bragança, Campus de Santa Apolónia, 5300-253 Bragança, Portugal; marianapedrosa@ipb.pt (M.C.P.); limalaires1@gmail.com (L.L.); sheleno@ipb.pt (S.H.); iferreira@ipb.pt (I.C.F.R.F.); lillian@ipb.pt (L.B.)

**Keywords:** metabolomics, secondary metabolites, bioactive, food safety, analytical chemistry

## Abstract

Secondary metabolites are molecules with unlimited applications that have been gaining importance in various industries and studied from many angles. They are mainly used for their bioactive capabilities, but due to the improvement of sensibility in analytical chemistry, they are also used for authentication and as a quality control parameter for foods, further allowing to help avoid food adulteration and food fraud, as well as helping understand the nutritional value of foods. This manuscript covers the examples of secondary metabolites that have been used as qualitative and authentication molecules in foods, from production, through processing and along their shelf-life. Furthermore, perspectives of analytical chemistry and their contribution to metabolite detection and general perspectives of metabolomics are also discussed.

## 1. Metabolomic Studies

### 1.1. Which “omic” Is It?

The great advance of genome sequencing technologies and studies of high-throughput in recent decades allowed the emergence of scientific areas that work on uncovering the complex biological systems at the molecular scale, such as molecular biosciences [1]. In 1990, the Human genome project (HGP) was established to map the human genome sequence, and in 2003, the project accomplished its goal, with the sequence being published and stored in the GenBank database. This database was created by The National Center for Biotechnology Information and holds DNA sequences from around 105,000 organisms, and the amount continues to grow [2]. The sequencing of the human genome has ushered in a new age in genomic studies, with the enhancement of techniques, the emergence of new technologies, the production of large amounts of data, and the development of bioinformatics [3,4]. 

Since the sequencing of the human genome, the studies of omic technologies have grown due to the pursuit of comprehension of biological mechanisms and functions at different cellular levels. The suffix “omic” in word indicates the studies of molecules such as genes, transcription, proteins, and metabolites with the purpose of better understanding biological systems in an integrated approach, investigating the processes involved, the response mechanisms, the products generated, the functions performed, the factors that influence the system, etc. The most notable examples are genomics, transcriptomics, proteomics, and metabolomics [3,5].

Dettmer et al. [6] described the “omic cascade”, with genomics indicated as the first level of the cascade, followed by transcriptomics, proteomics, and finally metabolomics. The collection of data from each level of the cascade may constitute a complex database for understanding the response of biological systems to different perturbations (e.g., environmental, genetic, disease-related). Metabolomics is at the end of the chain and represents the closest to a phenotype of cells tissues or organisms; it also represents essential building blocks and is necessary for the comprehension of changes in biological systems [5]. In essence, the four goals of metabolomics are to identify, quantify, investigate the dynamics, and elucidate the mechanisms of metabolism, considering the metabolites and the biological systems [7].

Metabolomic “is the study of the metabolite profiles of all the small-molecules in a living system”, providing a snapshot of the organism’s metabolic state and allowing for a better understanding of the metabolic pathways, their influences (e.g., health, diet, environmental factors), and impacts. Regarding metabolomic studies, there are other terms important to define and differentiate; “metabolome” is the complete set of metabolites in a living cell, tissue, organ, or organism [8,9,10] and metabolites small molecules with low molecular weight (<1–2 kDa) that are part of the metabolic reactions of an organism and essential for the maintenance of cellular functions. Due to the complexity, variety, and dynamics of metabolites, their analysis becomes an arduous and challenging process, usually requiring the application of different analytical techniques [3,5,6].

The metabolomics research can be divided into two main groups with some sub-divisions: (a) analytical technique, including sample preparation, extraction, separation, and detection with an appropriate method and data acquisition; and (b) data analysis, involving preprocessing, processing, and mining data according to the components of interest, as well as data treatment for visualization and storage to build a database [3,9]. One factor that has allowed the progress of omic studies is the advancement of analytical chemistry with more accurate and powerful equipment and techniques. Examples are chromatographic techniques, either liquid or gaseous, as well as capillary electrophoresis, which are widely used to fragment the components of the sample, which are subsequently identified by other techniques coupled to the equipment (e.g., mass spectrophotometer), enabling the quantification of metabolites. Furthermore, methodologies for analysis should provide an illustration of metabolites in a biological system at specific instants with adequate sensitivity and reproducibility.

Despite constant advancement, our knowledge is still limited, and more research is needed to understand metabolic pathways, identify individual metabolites, and integrate the information to build a metabolic network model that may predict the dynamics of biological systems as closely as possible [9].

### 1.2. Applications of Metabolomics

The application of metabolomic knowledge includes biomarkers, disease mechanisms, and new drugs [11] in different fields, namely medicine, agriculture, environmental sciences [12], and food science [13].

Metabolomics is a tool that can be used to accelerate the process of drug discovery and development, and it is useful in several steps for drug validation, namely in the identification of the responsible metabolite or biomarker, identification of the compound in chemical mixtures, study of the metabolism of the drug itself and the possible effects in the organism (e.g., toxicity and efficacy) [14]. Biomarkers can be useful to identify the presence of pollutants or to study climate changes and relate them to the effects on the biological system of an animal or plant. Campillo et al. [15], citing Wu and Wang [16] and Zhang et al. [17] regarding the application of metabolomics, studied metabolic responses in marine bivalves due to the presence of contaminants, such as lead, and revealed that the presence of this contaminant might cause neurotoxicity, osmotic, and energy metabolism dysregulation. However, the author also highlighted the possible interdependence between environmental stressors and pollutants in the results. In his article, he studied the effects of the contaminant polycyclic aromatic hydrocarbon fluoranthene in mussels and concluded that environmental stresses, in this case, nutritional stress, may cause similar changes in metabolic concentrations as pollutants.

Regarding medicine, metabolomics can be useful to identify the mode of action of new drugs by analyzing biochemical pathways and, in conjunction with complementary assays, identify therapeutic potential [18]. It can also be applied to understand how diseases and/or health problems act on organisms, the effects of drug use, and drug development and to identify responsible biomarkers [19]. It is important to note that the information generated through research contributes to the complementation of medicine and related areas; epidemiological databases are just one example [19].

Environmental metabolomics investigates natural and anthropological effects on metabolism through disturbances in the environment, analyzing the biochemical impacts of xenobiotics and diseases (e.g., differences in metabolite concentrations) on metabolic processes and relating these to variations in biological functions [15]. Cramer et al. [20] evidenced an increase, from 2001 onwards, of abiotic stresses (e.g., water, temperature, light, chemical, CO_2_, and NO_x_ oxide) on biologic systems and how this may impact agriculture and limit crop production. The authors highlighted the dynamism and complexity of metabolic responses, which can vary according to the duration, type and level of stress, type of cell, plant species, and other factors. Furthermore, they exemplified how water scarcity can inhibit growth and how reactive oxygen and nitrogen species may alter enzyme activity and gene regulation, affecting plant sugars; they also conducted several studies relating key aspects of abiotic stresses and the effects on metabolic responses of plants. A multi-targeted approach concerning “omics” is essential to understand the regulatory pathways and impacts related to environmental stresses, thus allowing a consistent identification of desired molecules and the construction of key regulatory network models for further biotechnological applications involving agriculture. Involving a different point of view, Almuhayawi et al. [21] investigated the consequences on Alfafa sprouts (*Medicago sativa* L.) of rising CO_2_ levels and reported that increased CO_2_ provided an increase in photosynthetic processes, boosting the production of nutrients (fats, fibres, carbohydrates, and proteins), minerals, and bioactive compounds, such as vitamins, phenolics, flavonoids, and antioxidant metabolites, favoring antioxidant and anti-inflammatory capacity of the vegetable. The author also evidenced the plant’s potential as a functional food. When thinking about global warming and climate change, it is often associated only with negative changes. However, the previous study demonstrated the importance of investigations of metabolic processes from various perspectives, highlighting ways of obtaining benefits from the problem of rising levels of CO_2_.

In the food field, metabolomics is related to the food system and can be involved in food safety, quality, and composition studies, in investigations of bioactivity of food metabolites and the relationship between diet and human health. The food product, together with its entire production chain (cultivation, manufacturing, storage, preparation, and consumption), comprises a complex set of systems, and to have a deeper comprehension of these systems, multidisciplinary approaches are required. Multifactor analysis and analytical techniques are tools to unravel the complexity of these interactions and their impact on food quality and safety [22,23].

## 2. Metabolomic Studies in Foods

### 2.1. General Considerations

In recent years, food metabolomic research has been growing due to the importance that food has for the economy and mankind [24]. The challenges in the food industry are constant and evolve with the new demands from consumers, such as more natural, healthy, and sustainable foods. In this sense, the industry must adapt to provide food with quality and safety, even though adversities in the face of climate changes, variation in the availability of resources, and means of production are ever-growing [23]. The global trend towards a plant-based diet, in addition to studies showing beneficial effects for health and the environment, is one example where metabolomics can be applied to ensure the quality of food for consumption. In plant-based products, the sensory characteristics are strongly related to the presence of secondary metabolites. Among these features, the flavor is the attribute that stands out the most and encompasses a set of several compounds associated mainly with taste and scent. Generally, the taste is linked with primary and secondary non-volatile metabolites (e.g., organic acids, polyphenols, alkaloids, tannins, and peptides), while the aroma is strictly linked with volatiles (e.g., esters, alcohols, aldehydes, terpenoids, and apocarotenoids). Therefore, the evaluation with omic technologies of the biochemical profile of fresh plant-based products regarding flavor metabolites presents itself as a potential instrument to measure the freshness and quality of the product, as well as to help build the path for further shelf-life, nutritional, and economic stability assessments [25].

The constant advance of omic technologies and studies, with greater integration of analysis between different molecule profiles, has driven the emergence of new omics strands, such as “foodomics”. This term, which emerged at the beginning of the 21st century, encompasses studies related to nutrition and food analysis with an omic approach [26,27]. Traceability, safety, quality, authenticity, functionality, toxicity, presence of contaminants, and impact on human health of food products are issues that can are investigated by foodomics [28]. Within metabolomics and foodomics, understanding secondary metabolites is essential to study their impact on food, health and how they can be used to ensure food safety, authentication, quality, and characteristic nutritional aspects.

### 2.2. Primary and Secondary Metabolites

As explained in Section 1.1, metabolites are small molecules related to metabolic reactions. Metabolites can be classified spatially as endogenous and exogenous, the former being derived from biochemical processes (e.g., carbohydrates, lipids, amino acids, fatty acids, or vitamins, polyphenols, and alkaloids) and the latter from xenobiotic metabolites (e.g., drugs, pesticides, pollutants, toxins, and food constituents). Another important classification is the provenance of the metabolite in relation to the metabolism that produces them; it is divided into primary and secondary [19,29].

Primary metabolites (PM) are molecules that act in the fundamental activities of organisms (growth, development, and reproduction) and are present in all types of organisms. Some examples are amino acids, nucleotides, vitamins, ethanol, organic acids, and sugars, among others [8,30,31]. Secondary metabolites (SM) are molecules that participate in the protective functions of the organism and do not play a direct role in the essential processes of growth, reproduction, and development of the organism, e.g., phenolic compounds, terpenoids, alkaloids, bacteriocin, and polyketides, among others. Although these molecules are produced in minor quantities in relation to the primary metabolites, they are natural compounds of great value and quite diverse in terms of structure, being plants, fungi, and bacteria the producers of these molecules [8,32,33,34]. Approximately 350,000 secondary metabolites, which are synthesized by plants, mostly phenolic compounds, and 70,000 by microorganisms have been identified [35]. Regarding the metabolites of microorganisms, only a small fraction of them have practical applications and are used in pharmaceuticals, agriculture, and other fields [36]. SM are usually produced in higher quantities under stressful situations, such as environmental adversities, nutrient shortages, or limited resources for growth [37].

**Figure 1:**
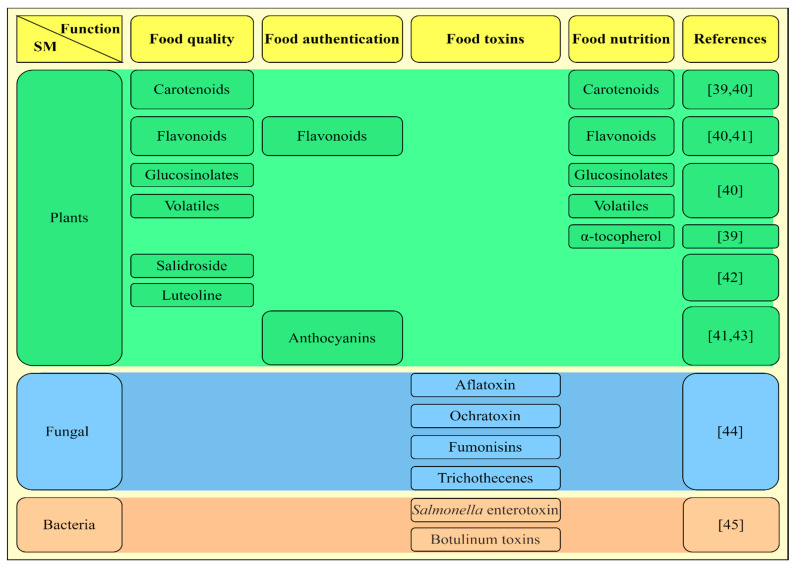
Depiction of some examples of applications of secondary metabolites from bacteria, fungi, and plants in metabolomics, mainly as biomarkers, found in previous studies. The same metabolite may have different functions in the food field, for example, as an indicator of quality in the processing and as a nutritional component. In addition, the metabolites of microorganisms are mostly indicative of toxins and food contamination [39,40,41,42,43,44,45].

The bioactive compound is another term widely used and related to secondary metabolites. They can be defined as SM, as they are not associated with the essential activities of cells. These molecules are widely present in the human diet, and their activity can provide various health benefits (e.g., prevention of cardiovascular diseases, cancer, diabetes, antiallergenic, and anti-inflammatory effects). Moreover, they are already widespread in the pharmaceutical and nutraceutical industries due to their bioactivity, and they are increasingly being applied to the food industry for different purposes [38]. Figure 1 shows a few examples of secondary metabolites from plants, fungi, and bacteria and possible application in the food field, mainly as biomarkers. In addition, the SM can act for different purposes in metabolomics studies.

Plant secondary metabolites are part of the plant defense system, acting to protect from the surrounding natural environment, namely ultraviolet radiation, insects, or other predators, especially due to plants being sessile organisms. This secondary metabolism operates as an intermediary in the relations among the biosystems and the plants [36]. Besides being an “armour”, it can also act on other fronts, such as signaling to attract pollinators and animals that can spread its seeds. Some examples of secondary metabolites are alkaloids, steroids, saponins, terpenes, tannins, flavonoids, and lignins, among others with bioactivity; phenolic compounds are the most prevalent ones in plants and synthesized from the pentose phosphate, shikimate, and phenylpropanoid pathways in plants [32]. The study of plant metabolites was one of the factors that helped boost the development of metabolomics due to the great variety and opportunities for research into plant biological systems. These metabolites can be applied in quality control, identification of food adulteration and contamination, as well as biomarkers [9]. They are valuable not only in the food industry but also in the pharmaceutical, cosmetic, perfume, and dyeing industries [46]. Secondary metabolites of plants can be applied in different fields of products, such as drugs, insecticides, flavorings, and colorants. These molecules are also a great part of the human diet providing color, flavor, and scent to the food [36].

Secondary metabolites derived from fungi throughout history have greatly impacted medicine in a positive way by saving lives. The prime and best-known example of secondary metabolites used in medicine is penicillin, discovered in the late 1920s. The pharmaceutical industry continuously searches for metabolites that can be used, and through research, several bioactive SM have already been discovered to inhibit the growth of fungi, bacteria, protozoa, insects, and parasites. Brief examples of SM are the aforementioned penicillin, cephalosporin (antibiotic), statins and cyclosporin (pharmaceutical products), and mycotoxins (toxic metabolites for humans) [47]. Other functions that should be mentioned are the cholesterol-lowering effect of lovastatin and immunosuppressive power of cyclosporine, among many others with life-saving bioactivities [36]. Therefore, these molecules present great potential for application in medicine, mainly for drug discovery and drug development and as agricultural chemicals. Fungi SM encompass a wide range of structures due to the need for survival in distinct habitats making fungi highly dependent on secondary metabolites. Thus, several of these organisms produce multiple types of secondary metabolites, and researchers point out that less than 10% of fungi have been investigated for their bioactive compounds. Some of these SM can have negative impacts, namely mycotoxins which can act as food contaminant, and in closed environments form molds, with a negative impact on food. Thus, the study of these substances becomes important to know and identify their bioactive pathway besides seeking ways to reduce their contamination [35,48].

Bacteria secondary metabolites biosynthesis occurs in the last phase of growth (stationary phase). These microorganisms are capable of producing multiple molecules with distinct biological functions, such as pigments, hormones, toxins, pesticides, immunosuppressants, antibacterial, and anticancer compounds [37,49]. Some examples are bacteriocins (antibacterial), siderophores (antibiotics), terpenoids, antimycins (fungicidal), geldanamycin (antibiotic), prodigiosin (pharmacological effects), and vancomycin (antibiotic). The vast majority of bioactive compounds from bacteria are produced by actinomycetes (phylum of Gram-positive bacteria) [34,50]. Bacteria SM can be applied in the pharmaceutical, agriculture, cosmetic, and animal feed industries. These SM are valuable for the development of new drugs for the pharmaceutical industry, acting with therapeutic properties against infectious diseases, cardiovascular diseases, and cancer. However, as well as in fungi, some metabolites may cause harmful effects on human and animal health, especially when related to food. Food pathogenic bacteria secrete toxins that can contaminate food products and processing water. These organisms become lodged in food products that have not been treated properly or have suffered damage (e.g., dairy products, canned, and packaged foods) and thus cause illness to the organism that consumes these products [37].

The functions of metabolites can vary in several areas and affect the human diet in different ways, both directly and indirectly. Hamacher et al. [51] reported the influence that condensed tannins might have when present in cattle diets, such as increased productivity, decreased methane (greenhouse gas) emissions, enhanced taste, smell and fatty acid profile of milk and meat, but they pointed out that these effects may vary with the concentration and presence of other bioactive compounds. Liu et al. [52] studied the relationship between browning in lettuce (*Lactuca sativa* L.) and the metabolic phenolic profile of the vegetable to identify possible biomarkers and apply them as a tool in the industry. The metabolites were analyzed by ultra-high performance liquid chromatography coupled with high-resolution mass spectrometry (UHPLC–HRMS) using a principal component analysis to analyze data. Twelve phenolic metabolites were identified as potential biomarkers for lettuce browning; caffeoylquinic acid, 9S,12S,13S-trihydroxy-10Z-octadecenoic acid, and caffeoyltartaric acid have already been reported in previous studies. The identification of biomarkers allows greater agility in the evaluation of lettuce browning. However, different production conditions should be evaluated to validate the markers. Pereira et al. [53] studied the effects of grapevine red blotch disease on *Cabernet Sauvignon* grape and wine quality in relation to primary and secondary metabolites. This virus may impact the metabolic pathways of grapevines, altering the production of secondary metabolites at ripening and the defense mechanisms of the plant, thus reducing them. The evaluation was done by employing two techniques, ^1^H NMR spectroscopy and reverse-phase high-performance liquid chromatography with diode array detector (RP-HPLC–DAD), specifically for the phenolic compounds profile, and statistically treated with analysis of variance, comparing the values by *t*-test and principal component analysis (PCA) for multivariate analysis. The results showed changes in the content of secondary metabolites in seed, pulp, and skin due to the presence of the virus. Zhan et al. [54] carried out a study, which identified and quantified 225 metabolites from residues of veterinary drugs and contaminants in raw milk samples by applying ultra-performance liquid chromatography and tandem mass spectrometry (UPLC–MS/MS). The research demonstrated the potential of this tool for monitoring and ensuring food safety in dairy processing

## 3. Platforms for Metabolomic Studies: Analytical Methods and Data Processing

### 3.1. General Aspects of Metabolomic Workflow

The two main pillars for the identification of structures of compounds in products are its purity and characterization of its elemental composition. For this purpose, analytical, chromatographic, and spectroscopic methods can be applied. The first relates to combustion and high-resolution mass spectrometry analysis to establish the elemental composition, considering molecular mass. The second is through the representation of peaks in chromatograms obtained from chromatography techniques. Additionally, the last one is by means of spectra obtained by absorption of infrared and ultraviolet spectrum and integration into the nuclear magnetic resonance [55]. Molecular analysis is one of the cornerstones involving studies for food safety, authentication, and quality [56].

Within the methods used for metabolomics, the analytical analyses can be grouped in fingerprinting when it is intended to determinate a pattern of metabolites in the sample without necessarily being interested in identifying and quantifying the components, and profiling, which usually consists in identifying and quantifying as many metabolites as possible [13]. The first approach generally aims to compare profiles according to variations in the surrounding ecosystem, whether environmental, genetic, or disease-related, while the latter studies a specific pathway or set of metabolites [57]. Related to that, the analyses may be targeted or untargeted; the targeted analyses the metabolites are already defined and known, whereas in the untargeted analyses, the objective is to gather all the metabolomic information of the sample, defining at a later point the desired data for the statistical analyses. The mixture of these two approaches may be an option to obtain complementary and more complete results [25,29]. Choi et al. [58] applied metabolomics with both approaches (targeted and untargeted) as well as LC–MS and GC connected to a flame ionization detector (FID) techniques to evaluate the quality and composition of coffees from Asia, South America, and Africa, according to the region of origin. Untargeted analysis was employed to obtain an overview of the metabolic profile of the samples and targeted for the quantitative analysis of metabolites and, in the end, used a PCA was applied for statistics. The authors pointed out the potential of integration between analyses to identify coffee quality in relation to the origin.

**Figure 2:**
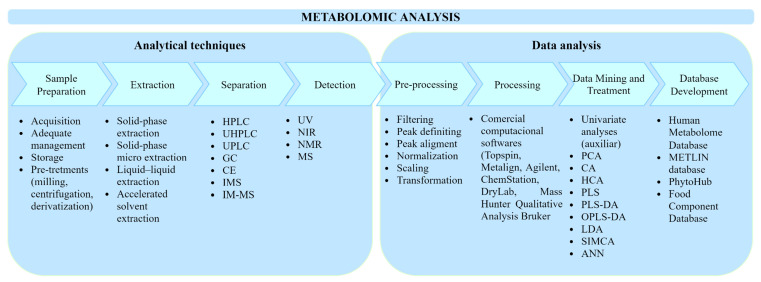
Basic workflow of metabolomic analysis.

The workflow of metabolomics studies can be divided into a few steps (Figure 2). However, not all are necessary, depending on the selected techniques. As there is more than one possible approach, defining the experimental design according to the research purpose is the first essential step for a satisfactory analysis [57]. Sample choice and preparation, as well as the extraction and detection method, are also initial factors that will determine the triumph of processing and comparison in data analysis and, consequently, the final result [9].

Sample preparation should consider acquisition, storage, and extraction. In the first two aspects, if not done correctly, metabolic changes may occur and mask, alter, inhibit, or overexpress specific metabolites, reducing the reliability of the results. The latter is critical for the course of the analysis because from it, the compounds of interest will be isolated from the specific matrix to be subsequently analyzed by the detection apparatus. In certain cases, pre-treatments are applied to concentrate the sample (e.g., milling, lyophilization, and microextraction techniques), volatilize the sample when analyzed by gas chromatography (e.g., derivatization), or remove undesired solid particles (e.g., centrifugation) [57,59]. Separation in the food field is mostly done by liquid chromatography (LC), high-performance liquid chromatography (HPLC) and ultraperformance liquid chromatography (UPLC), gas chromatography (GC), or capillary electrophoresis (CE) due to the wide range of molecules identification and resolution that can be obtained. Detection can be carried out by ultraviolet light (UV), near-infrared spectrometry (NIR), mass spectrometry (MS), or nuclear magnetic resonance (NMR). In addition, emerging technologies such as ion mobility (IM) and imaging mass spectrometry (IMS) are becoming tools to complement existing methodologies. However, the most commonly used in food analysis are NMR and MS. The combination of these techniques is widely used, the detection is coupled to the separation equipment, resulting in GC–MS, LC–MS, UPLC–MS, and HPLC–UV [24,42,59,60,61,62,63].

After data acquisition, the next steps relate to the multi-dimensional data analysis. Pre-processing is important to “clean” the data obtained in the detection to narrow down the most relevant information and “optimize” the data analysis [64]. This process may include noise filtering (removal of signals from the equipment and the experimental procedure), peak definition (identification of metabolite signals) and peak alignment (correction of possible peak deviations), normalization (removal of small variations caused by the method that are usually constant in samples), scaling (adjustment of the intensity of different variables), and transformation (adjustment of the distribution of the data and mitigating large outliers) [7,65].

The large amount of data generated by metabolomic studies demands the application of multivariate statistical techniques for its processing, enter Chemometrics. This field of chemistry applies “mathematical and statistical methods to extract relevant chemical information and to correlate quality parameters or physical properties to analytical data” [66] and a hot topic research area that has been growing, especially in the food sector [59,67]. In metabolomics, univariate analyses, such as analysis of variance (ANOVA) and *t*-test, can be used as auxiliary tools, indicating significance in variations between metabolites from different samples and further delimiting the variables for multivariate analyses [57]. Regarding multivariate analyses, they can be supervised when information about the groups of interest in the sample is already known, or unsupervised, which are exploratory techniques. The main examples of the former are partial least squares (PLS), partial least squares discriminant analysis (PLS-DA), orthogonal projections to latent structures discriminant analysis (OPLS-DA), linear discriminant analysis (LDA), soft independent modelling of class analogy (SIMCA), random forests (RF), and artificial neural network (ANN) [64,65]. The latter rely on PCA, cluster analysis (CA), hierarchical clustering (HCA), and *t*-distributed stochastic neighbor embedding (*t*-SNE) [7,60]. The treatment for visualization of the outputs is important to facilitate the understanding and relationship of the results, and, finally, the results obtained should be critically analyzed for inclusion or not in databases [9].

### 3.2. Separation Techniques

#### 3.2.1. Gas Chromatography (GC)

GC is a chromatographic separation technique capable of analyzing complex matrices and in which a gas constitutes the mobile phase. Briefly, the equipment’s working principle is based on sample volatilization and transport by a gas flow through a specific column until detection. The column is composed of two phases: the mobile phase, usually hydrogen or helium gas; and the stationary phase, which can be a porous, non-porous solid, or a liquid that can retain the substances for identification. Separation of the components occurs along the column with the passage of the mobile phase. In each partition of the column, after reaching equilibrium with the stationary phase, a portion of the solute is retained in the partition, and the remaining part continues to be transported by the mobile phase to the next segments of the column and the process repeats. Equilibrium is reached according to the solubility of each component at the column temperature [68,69,70,71].

GC is a technique with good fragmentation reproducibility, sensitivity, and potential for reliable identification and is mainly applied to identify volatile, gaseous compounds [14,72], as well as polar and non-polar metabolites with thermal stability. Some examples of compounds analyzed by GC are lipids, sugars, amino acids, and phosphorylated metabolites. Disadvantages of GC compared to LC is the need for one or more derivatization steps to cause thermal stability and volatility to prepare the sample, which can lead to errors and variations, hampering data comparison and impact on reliability [29,73]. Beleggia et al. [74] investigated the effects on the metabolic profile in the industrial processing of five pasta products. GC–MS and LC–MS were applied for metabolic profiling while ANOVA, PCA, and factor analysis were the statistical classifications used, identifying a total of 76 metabolites. The results showed that processing could negatively affect the quality, health benefits, and nutritional value of pasta due to temperature degradation and processing time of phytosterols, hydroxy fatty acids, tocopherols, and carotenoids. The control of the production parameters, as the use of lower temperatures, are possible solutions based on the results of the metabolomic studies. Therefore, this highlights the importance of such studies to develop the quality, understand the nutritional value, and ensure safety of food products.

#### 3.2.2. Liquid Chromatography (LC)

The separation principle of LC is basically the same as GC (equilibrium between the solute in the mobile phase and the partitions of the column and retention of the different analytes), but the main change is that the mobile phase is a liquid. LC is a well-known and widely used technique by research and industry, it can perform target and non-target analyses and is also capable of separating complex systems and components with different molecular weights and polarity, such as food matrices [56,75]. The stationary phase can be composed of silica, alumina, polar-bonded silica, or nonpolar-bonded silica. Regarding the mobile phase, there are innumerous options; however, the solvent choice must be in accordance with the performance of the instrument and allow suitable retention and separation of the solute in the column for analysis [75,76].

The great versatility of separation, sensitivity, resolution, and consistency and great range of identification of metabolites turn LC coupled with MS into the major technique applied in metabolomics [72]. This conjugation is capable of analyzing different compounds, such as terpenes, steroids, flavonoids, amino acids, dyes, and contaminants, besides being very efficient to investigate other plant secondary metabolites and food composition [9,56]. The disadvantages of LC are the need for high volumes of grade solvents, therefore increasing the cost of the process. Secondary metabolites are preferentially analyzed by LC due to their size, due to some becoming too large after sample preparation to be analyzed by GC techniques [73]. Additionally, due to the thermal instability and polarity of organic compounds [56], only a mere 20% can be analyzed by GC without any pre-treatment [77].

Advances in LC techniques, such as HPLC, UPLC, and UHPLC, in addition to the evolution of columns, allow faster and more efficient analysis of complex mixtures, favoring, for example, the identification of contaminants in food with greater agility [13]. HPLC is already a widely used technique to identify anthocyanins, flavonols, flavanols, and hydroxycinnamic acids in seeds, grapes, wine, and plant extracts [53]. In relation to food applications, it can act in the identification and characterization of biomarkers associated with contaminants, origin, processing, and assisting in quality control and authenticity [56]. Cubero-Leon et al. [78] studied the metabolome of carrots (*Daucus carota* L.) according to production system and region of origin, applying LC–MS and OPLS-DA as techniques. The OPLS-DA model was able to distinguish the differences between conventional and organic production of carrots and predict the region of origin of the vegetable. Secondary metabolites related to plant defense, along with metabolites related to carbohydrate metabolism, were biomarkers responsible for the differentiation.

#### 3.2.3. Capillary Electrophoresis (CE)

Despite the fact that CE is not a chromatographic technique, it is an analytical method for the separation of complex mixtures [79]. The operation of the CE is based on the application of a potential difference, generating an electric field in which the ions move through the capillary. Thus, charged substances, such as ions (cations and anions), migrate to the positive or negative pole according to the cargo. The vast majority of capillaries used are made of fused silica [80] and filled with buffer solution to allow separation [81]. The separation is done according to the mass-to-charge ratio (*m*/*z*), and the components must possess a charge for separation to occur. Although it has a better separation efficiency than HPLC, it has a lower sensitivity [82]. It is an underestimated technique in relation to chromatographic techniques, which can be explained by the greater consolidation of chromatographic techniques in the market and by the belief that CE presents less reproducibility, which can be overcome with improvements in the internal surface of the capillary [79]. Advantages of this technique are the low cost of consumables, low sample volume, and simplicity of the equipment, and the peak intensity is related to the concentration of ions in the sample, allowing quantification on a direct basis. The main limitation is the need for the analyte to be charged, but it can be overcome with specific treatments [79,80].

As in GC and LC techniques, in CE, the union with MS enhances the application and has shown growth in recent years [81]. Metabolomics CE-based studies in the food field are widely in connection with metabolite profiling, and despite identifying secondary metabolites, the main findings and biomarkers are still related to primary metabolites [83], for example, to identify potential biomarkers to differentiate conventional and transgenic soybean [84] and to differentiate conventional and transgenic maize. Both studies used capillary electrophoresis time-of-flight mass spectrometry (CE-TOF-MS) [85].

#### 3.2.4. Ion Mobility–Mass Spectrometry (IM–MS)

IM is gas electrophoresis, which separation principle of this technique occurs according to the characteristics (charge, size, and shape) of the ions generated due to ionic collisions that happen in the medium. IM–MS technique has emerged in recent years with the aim of improving the analysis of metabolites, in a fast way, also in the structural and mass aspect [86]. It is a separation technique with low separation time (in the millisecond range) and can be a tool employed to increase the reliability in the identification of non-targeted metabolomics studies, especially when coupled with the MS method [87]. The union of IM–MS with other chromatographic techniques (e.g., LC–IM–MS) enhances the multidimensional separation while maintaining the same dimensional range [63]. Being a relatively new technique in metabolomics applications, there are still some challenges related to software capacity for data extraction, calibration, database construction, and standardization. However, it presents great potential for metabolomic studies [86].

### 3.3. Detection Techniques

#### 3.3.1. Nuclear Magnetic Resonance (NMR)

NMR is a popular and widely applied technique in metabolomic studies, and in the last two decades, it has been rising alongside MS. NRM is a powerful technique that can be applied in liquid and solid matrices with versatility and is able to identify and characterize metabolites in complex systems such as food [88,89]. The working principle of the technique is based on the magnetic properties existing in atoms with odd atomic numbers. Due to the odd number, a nuclear spin is generated, creating a magnetic moment. Thus, when an external magnetic field is applied by NMR, an interaction occurs between them and the equipment is able to extract information about the sample [89,90]. The elemental composition of food generally shows at least one isotope of H (hydrogen), C (carbon), N (nitrogen), P (phosphorous), or O (oxygen) that can be identified by NRM, reinforcing the suitability of the technique for food analysis [91,92]. Advantages of this method are reproducibility and non-destructibility, allowing an analysis of the integral sample, as well as robustness and low downtime of the instrument. The main disadvantages are few software resources, small spectral databases, overlapping peaks, and low sensitivity [11,88] with a required concentration range between micro and millimolar, and a considerable investment in equipment and technician training. Compared to mass spectrometry methods, NMR is about 50% less sensitive; however, it is advantageous in the sense that it has methods with lower operating cost, mainly due to the low cost of sample preparation, allowing direct and quantitative correlation between molar concentration and the magnetic resonance values [12,73].

Due to these advantages and evolution (e.g., higher magnetic fields, cryogenic probes, and automation), NRM is gaining space in the food science field for authentication, safety, quality, sensory evaluation, nutritional composition, and interaction with human health, such as in beverages, meats, oils, vegetables, and other food products [89,90]. Moreover, despite being high-cost equipment, it is the technique seen as the most viable for large-scale applications compared to MS-based techniques [91]. Metabolomics technology based on ^1^H NMR is already applied and commercialized for wine authentication and quality. Amargianitaki and Spyros [43] reviewed NMR metabolomic approaches, including examples of secondary metabolites, to obtain data on different aspects of wine production (cultivar, geographical origin, and vintage). The authors cited the evaluation of anthocyanins (e.g., peonidin-3-glucoside, petunidin-3-glucoside, cyanidin-3-glucoside, delphinidin-3-glucoside, and malvidin-3-glucoside) with the application of 1D and 2D NMR for authentication in Slovenian red wines against the presence of black rice anthocyanins, as well as the union of LC–NMR and LC–MS to identify the composition of four different varieties of grapes. Another application was the use of ^1^H NMR spectroscopy and PCA score plot to differentiate the starter fermentation of white wines. In the end, the authors highlighted the importance of the integration between NMR and analytical techniques, such as LC–MS and GC–MS, to obtain more complete wine metabolomic profiles, including phenolic, aromatics and volatiles, and tannins, among others. Rocchetti and O’Callaghan [12] point to ^1^H NMR as an efficient tool to trace metabolites in milk, namely organic acids, vitamins, aromatic compounds, and nucleotides.

#### 3.3.2. Mass Spectrometry (MS)

MS is one of the analytical techniques that exhibits the largest growth in publications in recent years, and the food sector is a major beneficiary of this growth. The working principle of MS considers three stages: conversion, characterization, and detection. The equipment converts the sample into gaseous ions by ionization, which are then separated by a mass-to-charge ratio (*m/z*) in a mass analyzer and, in the end, are detected and recorded by a detection system according to the amount of each ion species [93,94]. Despite the advantages of using NRM, in the dispute between NRM and MS detection methods, MS usually comes out ahead as the choice in most metabolomic studies. The main reasons are the higher sensitivity (identification at nanoscale), wider detection range, powerful molecular structure detection, large spectral database, greater variety of available software, and smaller equipment footprint [7,88,91,94]. Nevertheless, it is a method that has disadvantages, such as being destructible to the sample, lower reproducibility, complex sample preparation, more fragile instrument, with longer downtime, and the need to use chromatographs, and the quantitative correlation is not direct with the intensity obtained, depending on the efficiency of ionization [88,91].

The combination involving chromatographic techniques (detection) and mass spectrometry (separation) contemplates an effective tool for the analysis of complex matrices as is the case of foods, characterizing the structure and mass of molecules in a robust and precise manner [65]. Mamat et al. [61] gathered the GC–MS and LC–MS techniques to evaluate the metabolic profile (primary and secondary metabolites) in mangosteen (*Garcinia mangostana* Linn.) at different times of ripening. Fruit ripening is a phenomenon that can change the texture, nutritional profile, flavor, and coloration due to biochemical and physiological processes. GC–MS was able to identify 57 metabolites and LC–MS, 98 metabolites. Regarding secondary metabolites, the application of variable influence on projection (VIP) (≥1.00 and *p* ≤ 0.05) pointed out the greatest influence of the following compounds: neoisostegane, epirobinetinidol-(4β,8)-catechin, α-mangostin, and gartanin in the ripening stages. The metabolomic results are a source of information for producers and for solution development to expand the shelf life of mangosteen in the market. Lee et al. [95] studied the presence of markers to differentiate the herbal *Schisandra chinensis* herb according to their origin from China and Korea with the help of metabolomics. LC–MS and GC–MS were applied for primary and secondary metabolites analysis and PCA for multivariate statistical analysis. The study pointed out nine secondary metabolites (omisin D, tigloylgomisin P, gomisin G, angeloylgomisin P, schisantherin C, gomisin B, gomisin F, benzoylgomisin O, and benzoyl isogomisin O) and three primary metabolites (1,2,3-propanetricarboxylic acid, d-fructose, and galactose oxime) as biomarkers; the secondary metabolites showed more qualitative and quantitative values in relation to the discrimination performed.

### 3.4. Data Processing

After acquiring the analytical data, metabolomics proceeds to computational analysis, performing multivariate statistical analysis and comparing characteristics, in addition to evaluating and relating to existing data in metabolomic databases [28,96]. The treatment transforms the outcomes into discernible data, synthesizing key findings by transferring large amounts of analytical data into discrimination maps, score and loading plots. The identification process uses existing specific databases as a resource for comparison according to the fragment ions obtained in the assay [12]. Table 1 presents a set of metabolomics databases involving metabolomic pathways, general and specific metabolites, spectra, molecule characterization, etc.

Comparing data is still an arduous and challenging process [14], although in the market, there are different available tools to process data for use in MS and NMR. However, the outputs tend to vary according to the used software, impacting the results and hindering the comparison between the data [12]. Multivariate data analysis is popular to manage data from untargeted metabolomics studies [14]. The application of PCA is an option for drawing first conclusions from a group of samples, such as the main differences, due to the exploratory nature of the analysis, while also being a methodology widely used for classification and comparison of samples. Reid et al. [99] studied the adulteration of strawberry purées, in which apple purées were added to “complement” the product. The authors used solid-phase microextraction with gas chromatography and chemometric data analysis by means of a PCA to differentiate the constituents present in the sample. The methodology proved to be satisfactory to identify adulteration, especially at higher concentrations. Moreover, it was possible to identify the reference compounds for the adulteration analysis, excluding compounds that were not essential and favoring a more specific statistical analysis. Although the studies present the potential of using this technique, namely for food authentication, further research is still necessary for its improvement, specifically to identify different varieties of apples and fermentation levels of strawberry puree.

PLS and OPLS analysis can assist in obtaining more accurate and robust data, validating the information from the exploratory analysis by amplifying the variability between samples [12,64]. LDA and PLS-DA are supervised statistical analyses that use latent variables to enhance the differentiation between classes. The first sets a direction and focuses on the distinctions between classes for the analysis. The second defines a matrix to perform the classification between samples. This strategy has already been employed to evaluate adulteration in lavender essential oils, both in relation to quality and to geographical origin, according to the chemical compounds involved, including secondary metabolites [64]. Pramai et al. [100] evaluated the relation of metabolites and biological activity by PLS-DA of three germinated rice varieties and one non-germinated standard, with the application of the metabolomic technique for separation and detection of H^1^ NRM. The results obtained demonstrated the separation of the varieties into three clusters, therefore allowing differentiation besides the correlation between bioactivity and presence of metabolites (a-linolenic acid, g-oryzanol, a-tocopherol, g-aminobutyric acid, 3-hydroxybutyric acid, fumaric acid, fatty acids, and amino acids) and the increase of phenolic content after rice germination. Regarding the metabolic pathway of biosynthesis analyzed, the increased production of secondary metabolites in the rice may bring benefits for the nutritional value and health.

## 4. Application in Foods

### 4.1. Food Safety

Food safety is a topic that has been growing over the years due to the greater ease with which regional products with specific characteristics can be marketed across the globe, as well as tighter regulations and consumer awareness. In this sense, this issue has become an essential aspect of attention for governments and regulatory agencies to protect the consumer and ensure quality. Regulatory agencies can act by defining and controlling what is and what is not allowed in food, both in relation to the prohibition of substances and the definition of a maximum admitted amount, as well as setting labeling standards to ensure safety. The development of MS-based metabolomics analysis is a powerful tool to assist in the detection of substances in food and for regulatory purposes [13,101]. In the European Union (EU), the regulation (EU) n° 1169/2011 has an objective targeted at developing principles, requirements, and responsibilities regarding food information, including labeling, aimed at consumer safety [102]. The EU has a series of initiatives to fight food fraud, such as the development of The Food Fraud Network and geographical indicators to ensure the specific quality that the consumer expects from the product, namely Protected Designation of Origin (PDO), Protected Geographical Indication (PGI), Geographical Indication (GI), Traditional Specialty Guaranteed (TSG), the product of EU’s outermost regions, and mountain product [103,104]. The Codex Alimentarius (part of the union between the Food and Agriculture Organization of the United Nations and the World Health Organization Food Standards Programme) also has a collection of standards and guidelines concerning the nutritional content and labeling of foods to protect and better enlighten consumers [105]. To achieve food safety, food quality and authentication are important aspects. Table 2 shows some examples of the application of metabolomics studies in the food field with such goals.

#### 4.1.1. Quality Control for Foods

Food quality is a factor that encompasses several product characteristics, namely taste, color, composition, smell, and other physical and chemical properties, making its analysis quite complex and challenging. Moreover, a single analytical technique is not enough to investigate all these factors simultaneously [13]. All stages of the food production chain can interfere with food quality. The final quality properties of a cultivated plant depend on its metabolome [9]. In green teas, for example, non-volatile metabolites are essential in determining the flavor quality and functionality of the tea. Flavonoid glycosides, caffeine, and catechins influence the bitterness and organic acids impact on the fruitiness and acidity. Carotenoids and fat-soluble chlorophylls have an effect on the physical parameters, such as the color and shape of the dry tea [108]. The quality of post-harvest plant-based products is also related to the evaluation of the presence of contaminants, microorganisms, heavy metals, and maximum levels of permitted substances [25]. Food processing is a dynamic process that transforms food (*e.g*., heating, mixing, drying, and fermenting) and may change its properties, such as texture, flavor, and aroma, and modify the nutritional profile. Therefore, once again, secondary metabolite analysis becomes a tool to evaluate and maintain a standard of quality and safety [23,24]. Lopez-Sanchez et al. [40] investigated the effects of industrial processes on metabolites in tomato, broccoli, and carrot purees in liquid and semi-liquid states. Metabolomics profiling targeted approaches applying HPLC coupled to photodiode array detector and GC–MS and untargeted approach applying ^1^H NMR for polar components, reversed-phase LC-PDA-QTOF MS for semi-polar and headspace solid-phase micro extraction (SPME) coupled to GC–MS for volatiles components. The author pointed out the perturbation in phytochemical profiles (carotenoids, flavonoids, glucosinolates, and volatiles) of vegetables only modifying the processing order, in this case between blending and heat treatment. Change is mainly driven by the activation of endogenous enzymes.

Quality control is important, because it is linked to the well-being and relations with the consumer, aiming at an suitable final product, both in relation to product safety and acceptance [25].

#### 4.1.2. Authentication for Foods

Food authentication is part of the process to ensure the veracity of the information described by the label (*e.g*., origin, production, processing, and nutritional profile), ensuring the safety and quality of the product according to the unique characteristics expected by the consumer. One of the main reasons for adulteration is for economic purposes, that is, to make the product cheaper, and the major forms are adding lower-cost or non-food components to high-value products, adding and/or replacing inferior level ingredients into high-grade foods, and changing the label with false information about the region or production method of the product. In this way, the advanced omic technologies appear as tools to counter food fraud [57,101]. For example, organic products usually have higher added value than inorganic ones due to additional costs in their production, such as certifications. The final value of the product can be up to 50% more expensive, and data indicate that in the USA, the organic products market should increase fivefold by 2025. Therefore, the growth of the market also boosts the increase of frauds. For combating such frauds, the use of metabolomic techniques based on high-resolution mass chromatography, including targeted analysis to identify secondary metabolites (e.g., phenolic compounds and organic acids) and untargeted metabolomic fingerprint analysis, are proving to be potential tools for such differentiation [72]. Studies related to food authentication are usually aimed at finding distinctions amongst samples of the same population (e.g., specific food product) (discriminative) and building statistical models that can predict effects due to the association of compounds facing different conditions (predictive) [57,109]. Metabolite profiling can act as an analytical fingerprint and may distinguish these differences from the existence of biomarkers. In plant foods, in which phenolic compounds (secondary metabolites) are widely present, they can serve as biomarkers due to their protective properties against external stresses. For instance, in the absence of pesticides, the plant tends to increase the production of secondary metabolites to defend itself. However, there is still a need for generalized research to validate such authentication method, as it is not a consensus in the scientific community, and metabolic profiles may vary according to other factors, such as genetics, environmental factors, and/or variations in climate [41]. Erban et al. [110] investigated through metabolomics and machine learning the presence of secondary metabolites as processing biomarkers of chia, linseed, and sesame in cookies. These seeds are considered as high-value food ingredients and defined as “superfoods” by the market. Untargeted GC–MS metabolomic profiling was the technique chosen, along with random forest analysis (machine learning technology) and PCA for data processing. Although the study pointed out the presence of 4 processing-dependent biomarkers for chia (4-hydroxybenzaldehyde, trisaccharide, methylinositol, and tyrosol) and linseed (succinic acid monomethylester, raffinose, and two unidentified compounds) besides linoleic acid and oleic acid as potential biomarkers for sesame seeds in cookies, it evidenced that the molecules found were not ideal as markers and may not be reliable for authentication. In addition, the author showed the need for future studies for the presence and variations of the biomarkers of these seeds in different processing steps and application of different analytical methods, such as LC–MS, to possibly identify other metabolites as biomarkers.

#### 4.1.3. Food Toxins

Microbial food safety is an important issue for the industry and of paramount interest to consumers. The various stages of the production chain can present fragile points for food contamination, namely cultivation, production, processing, storage, and distribution. Pathogens can settle in the product, develop, and produce toxins that are harmful to human health. In some cases, these toxins do not change the organoleptic characteristics but intoxicate only after ingestion. Bacteria, fungi, and algae are the main ones responsible for the production of these toxins. Therefore, integration between all parties to monitor and ensure food safety becomes essential. In this sense, “foodomics” becomes an ally for the prevention and monitoring of pathogens in food during the production stages [111]. Due to the negative impacts that food contamination can have on the organism, there is an increasing interest in fungal toxins in food and in the safety of food processing. Mycotoxins can appear in a wide variety of foods (e.g., vegetables, fruits, cereals, and animal products) and do not necessarily disappear after the complex production steps and are capable of being transferred to the final product. The metabolomics approach can be applied as a tool for the development and control of fungal contamination of food to enable a safe and quality food product. In this context, the identification of mycotoxin biomarkers is essential to understand the dynamics of these toxins and improve risk assessment in foods during processing. IMS is an example of a high throughput technique with potential for the detection and screening of these toxic metabolites in vegetables, fruits, and food of animal origin [44].

Furthermore, metabolomic studies can identify the metabolites produced in infections and biomarkers and help to understand the activity of microorganisms during food contaminations and disease development. Metabolomic datasets can guide to more accurate actions against contamination outbreaks and ensure food safety against microorganisms [112]. The presence of toxins deteriorates the quality of food and may cause adverse effects on human health. Fungi of the genera *Fusarium*, *Aspergillus, Monascus*, and *Penicillium* are some of the main responsible for the presence of mycotoxins in food. MS-based tools are techniques capable of identifying these substances [13].

### 4.2. Nutritional Value Assessment of Foods

Nutritional metabolomics studies the correlations and impacts of food, diet, nutrients, bioactive components, and microorganisms on biological systems associated with the metabolism of organisms [113]. In general, nutritional metabolomics studies differ from pharmacological ones in terms of the intensity of the distinctions between the analysis outputs. Nutritional metabolomics investigates disorders in the organism’s health and uses a control for comparison between the application of different diets (e.g., functional food or nutraceutical) or specific compounds and can evaluate according to the previous health status. However, the intensity of the differences between “subjects” are usually more discrete, while pharmaceuticals are more defined and visible [1,24]. LC–MS and GC–MS are the preferred techniques due to their sensitivity for experiments with specific diets and their effects on the individual’s organism [114].

One major goal of nutritional assessment is to evaluate the effects that certain compounds may have on the organism, the synergy between distinct compounds, and biomarkers to assess the relationship with health. These molecules can act as indicators of the body’s nutritional condition. The lack of molecules such as certain fatty acids, carotenoids, and vitamins can generate dysregulation in fat deposition, cholesterol levels, and insulin activity, relating to the onset of chronic diseases (e.g., obesity) [1,23,39], for example, assessing the influence of dietary patterns on the human body, such as plant-based, high meat, or fruit consumption and finding the responsible biomarkers relate to the intake [114]. Another evaluation that can be done is monitoring metabolites in the blood, urine, and saliva, using the same logic as tests for monitoring the presence of drugs in the body. This kind of monitoring can help the interpretation of the physiological and biochemical effects of metabolites in the body. In this sense, urine assessment is a better indicator than blood for the presence of biomarkers that are excreted by the body (e.g., phytochemicals, glycosylated, glucuronated, or sulfated conjugates) from specific foods [114]. For instance, the presence of salicyluric and salicylic acids in the urine indicates high consumption of fruit and vegetables and can be used to identify vegetarians as they consume more plant-based foods [115]. Solanky et al. [107] investigated the intake of soy isoflavones by premenopausal women and reported an influence on the individual’s energy metabolism and variations of substances in the plasma profile.

Toffano et al. [39] evaluated the dietary status in children and adolescents with the aid of the Brazilian Healthy Eating Index-Revised (BHEI-R) (index with nutritional recommendations) and the presence of biomarkers. The work pointed out the association of omega-3, omega-6 fatty acids, and β-carotene with fruit and vegetable intake, as well as the presence of retinol and pyridoxine with dairy intake. Such association allowed the evaluation of the diet quality of the population studied in relation to consumption of previous foods and concluded the precarious nature of the diet of children and adolescents, with low ingestion of fruits, vegetables, dairy products, and grains. In addition, it obtained as an important conclusion the validation of the index together with biomarkers as tools to assist future metabolomics and nutritional studies.

### 4.3. Food Processing

One objective of food processing is to modify and transform raw foods (e.g., vegetables, fruits, meat, and milk) into products with higher added value and to promote safety through techniques, such as fermentation, freezing, heating, mixing, pressing, and others. During the processing steps, the organoleptic characteristics, besides the nutritional profile related to the metabolic profile of the food, may change and affect the final attributes of the product. Moreover, not only processing but also transportation, storage, and packaging are processing-related steps that can impact product characteristics and quality. Therefore, monitoring and assessing with metabolomics techniques are potential tools to ensure quality throughout the food processing chain. In this sense, food safety and authentication are also involved in processing. Safety is vital to avoid possible contaminations occur during the process and transfer to the final product, preventing damage to consumer health [24,116,117]. Authentication can be employed to indicate the processing that food has undergone, as changes in the metabolite profile generated due to different treatments can provide characteristic profiles and allow identification of the processing and even the production site [118]. Monitoring can be done by identifying the deterioration of metabolites due to a specific process (e.g., freezing and heating), presence of freshness biomarkers [116], metabolomic profiles, and others. The monitoring can also be useful to optimize the processing conditions and analyze the lifetime of the products more efficiently [117].

Processing can be considered an enrichment of the product metabolome, providing complexity and data for authentication. In addition, monitoring can allow for greater standardization in product quality [119]. For example, Lucini et al. [118] identified distinctions in the phenolic profile of tomato products (crushed pulp, puree, and paste) and the difference in tomato paste under three different treatments (cold, warm, and hot). The authors observed the phenolic fingerprint between the paste treatments, especially in relation to the flavonoids, phenylpropanoids, and lignans, as well as between processing sites. Furthermore, they indicated the potential of the profiles as processing signatures for metabolomics studies. Table 3 exemplifies some studies with metabolomics application related to the presence of secondary metabolites in food processing.

## 5. Main Challenges and Difficulties

Although research is increasing, widespread, reliable databases are still limited. Moreover, another challenge is the diversity in chemical and physical properties of metabolites, hindering the simultaneous analysis of several metabolites by detection and identification techniques [6]. Recognizing the significant and relevant differences between different metabolic profiles [9] and construction of a model that allows predictable and reliable outputs from the obtained data is still a hurdle, especially for bioinformatics, due to the great complexity and variety of these data [1]. The main challenges for metabolomic analysis in the processing are the complex metabolite profiles, with compounds with different chemical classes and requiring different analytical methods in addition to a database that is still under construction [117]. For instance, post-harvest processing steps can impact the presence of biomarkers in products, adding additional variables and complexity to authentication analysis. The data set of the experiment presents itself as another challenge. The size of the study population must be defined to account for variations in origin, season, climate, species, variety, etc., in addition to being generalized enough for the application of the models to as yet unknown data. In general, metabolomics studies follow the same workflow, depending on whether certain steps are required or not. However, the work possibilities are diverse, as there are options for separation and detection methods, different analysis software, and distinct statistical analyses. All these factors contribute to the diversification of results [41]. Standardization of metabolomic analysis methodologies is another present challenge that is intertwined with the creation of reliable databases. Standardization allows the acquired data to be comparable between different studies and enhances the coverage of databases [57,111].

In nutritional, the same problem repeats itself due to a great amount and high dynamics of a metabolite concentration [1]. A large number of results are obtained in the spectrum of metabolomics data, and identifying all metabolites is a challenge, together with the identification of metabolites that are specifically related to human nutrition [115]. Age, gender, genetics, and the different responses of the body to diet complicate the standardization of metabolomic profiles and comparison between databases, as well as the technological and database limitation, which still need to evolve to cover the identification of more details of metabolomic profiles [114].

To summarize, the main difficulties are, beyond reliable databases, which are still under constant construction and do not always allow comparison of results due to variation in methodologies, technologies for identification and quantification, as a single technique is not capable of identifying a complete metabolic profile and does not always have the appropriate resolution, and the complexity of the biological systems themselves. Furthermore, most studies do not just focus on secondary metabolites but on metabolites in general.

## 6. Trends and Conclusions

The creation of biosensors to identify secondary metabolites in relation to consumer perception is a potential novelty. Biosensors are devices that can assist in the evaluation of sensory qualities of plant-based products by means of detection of volatile metabolites. The main examples are electronic noses and electronic tongues, which through patterns of metabolite profiles of aromas and flavors can mimic some human sensory characteristics (taste and scent). In this way, they can assess metabolic impacts on the freshness and quality of fruits and vegetable products during storage and post-harvest. These biosensors can be applied in conjunction with GC–MS methodology to provide more integrated information on metabolites, both in sensory issues and to sample composition [25]. Furthermore, a potential new form of analysis in foodomic is the multi-block method, which covers not only the statistics across sample variables but the data set available between different analysis techniques, for example, between MS and NMR. Foodomics involve complex interactions and the application of more extensive multivariate analyses. In addition, analytical data are constantly increasing, with more research and an increasing number of comprehensive databases available. Therefore, the evolution of chemometrics becomes an essential factor in enhancing a more complete understanding of biological systems [28]. The combination of different identification techniques is also a trend to enhance the fields of study of complex biological processes [73].

Despite technological advances, the number of unidentified metabolites, mainly secondary metabolites, is still large and reduce the “anonymity” of these compounds is a constant challenge [73]. The evolution of metabolomics applied to food has evolved in recent years, which is evidenced by the increase of publications, as well as the appearance of new metabolomic strands, such as foodomics. Alongside this, the development of faster and more sensitive analytical technologies, chemometrics, bioinformatics, and the construction of more robust metabolomics databases has allowed the increase in the range of applications. By making these techniques more “accessible” to industry, it favors food authentication, control, quality, and safety in a world with global food trading and with more demanding consumers. The objective of metabolomics is to discover markers and then make them useful in several sectors, improving traceability, quality, security, and safety. Despite increasing research, secondary metabolomics still has great potential for innovation; it is just a matter of how much mankind is willing to invest to further tap into their inexhaustible potential.

## Figures and Tables

**Table 1:** Examples of some online omics databases.

Metabolites Databases	URL *	References
Kyoto Encyclopedia of Genes and Genomes (KEGG)	http://www.genome.jp/kegg/	[14]
Human Metabolome Database (HMDB)	https://hmdb.ca/	[14,29]
Lipid Maps	https://www.lipidmaps.org/	[14]
METLIN database	https://metlin.scripps.edu/landing_page.php?pgcontent=mainPage	[14,29]
BioCyc	https://biocyc.org/	[14]
Spectral Database for Organic Compounds	https://sdbs.db.aist.go.jp/sdbs/cgi-bin/cre_index.cgi	
Lipidblast	https://fiehnlab.ucdavis.edu/projects/lipidblast	[29]
MassBank of North America	https://mona.fiehnlab.ucdavis.edu/	
Food Component Database	https://foodb.ca/	[24]
PhytoHub	https://phytohub.eu/	[23,24]
PhenolExplorer	http://phenol-explorer.eu/	
CHEMnetBASE	https://www.chemnetbase.com/faces/search/SimpleSearch.xhtml	[35]
Milk Composition Database (MCDB)	https://mcdb.ca/	[12]
Bovine Metabolome Database (BMDB)	https://bovinedb.ca/	
BiGG Models	http://bigg.ucsd.edu/	[97]
SetupX and BinBase	https://fiehnlab.ucdavis.edu/projects/binbase-setup	
SYSTOMONAS	http://www.systomonas.de	
MetaboLights database	https://www.ebi.ac.uk/metabolights/	
MetaCyc	https://metacyc.org/	
HumanCyc	https://humancyc.org/	
BioCyc	https://www.biocyc.org/	
Reactome	https://reactome.org/	
WikiPathways	https://www.wikipathways.org/index.php/WikiPathways	
Chemspider	http://www.chemspider.com	[98]
PubChem	https://pubchem.ncbi.nlm.nih.gov	
Knapsack	http://kanaya.naist.jp/knapsack_jsp/top.html	
Biological Magnetic Resonance Data Bank (BMRB)	https://bmrb.io/	[91]
NMRShiftDB2 database	https://nmrshiftdb.nmr.uni-koeln.de/	
AIST spectral database in Japan	https://sdbs.db.aist.go.jp	

* All sites were accessed on 22 July 2021.

**Table 2:** Examples of metabolomic approach (authentication, quality, and nutritional) in foods.

Food Purpose of Analysis		Detection Technology	Data Treatment	References
Authentication	Milk compounds (sugars, vitamins, nucleotides, and aromatic compounds) to distinguish milk from pasture and indoor total mixed ration-based diets	^1^H NMR	PLS-DA	[12]
Authentication	Variation of coffee components by region	LC–MS and GC-FID; targeted and non-targeted analysis	PCA	[58]
Authentication	Identification of anthocyanin content of red wines to detect possible adulteration with black rice anthocyanins	^1^H NMR and Fourier transform near-infrared		[43]
Authentication	Identification of anthocyanins in *Pinot Noir*, *Cabernet Sauvignon*, and *Merlot* red wines	NMR and LC–MS		
Authentication	Anthocyanins profile of grape berry skins belonging to different grape varieties	HPLC–MS and HPLC–NMR		
Authentication	Analysis of the aromatic composition of wine phenolic extracts	LC–NMR/MS		
Authentication	Differentiation of *fiano di Avellino* white wines obtained by fermentation with either a commercial or a selected autochthonous *Saccharomyces cerevisiae* yeast starter	^1^H NMR	PCA	
Authentication	Metabolite profiling to study the fermentative behavior of lactic acid bacteria in grape wines	^1^H NMR and GC		
Authentication	*Negroamaro* red wines obtained through different wine-making technologies (traditional, ultrasounds, and cryomaceration with dry ice) and soil management practices (soil tillage and cover crop)	^1^H NMR and GC	PCA (unsupervised) and OPLS-DA (supervised)	
Authentication	Prediction of the origin of the agricultural system through metabolite profile of carrots (*Daucus carota* L.)	LC–MS; untargeted	OPLS-DA	[78]
Authentication	Determination of *Schisandra chinensis* herb origin through GC–MS and LC–MS, and primary or secondary metabolites	GC–MS and LC–MS	PCA	[95]
Quality	Identification of metabolomic marker compounds to predict lettuce (*Lactuca sativa* L.) browning	UHPLC–HRMS; untargeted analysis	PCA; SIMCA 13	[52]
Quality	Identification of metabolites in mangosteen (*Garcinia mangostana* Linn.) that contribute to ripening characteristics	GC–MS and LC–MS	PCA and PLS-DA	[61]
Quality	Influence of grapevine red blotch disease on the primary and secondary metabolites in skin, pulp, and seed tissues of *Cabernet sauvignon* grapes at harvest	^1^H NMR and RP-HPLC–DAD	PCA, analysis of variance and the two means of each variable, *t*-test	[53]
Quality	Identification of biomarkers compounds responsible for freshness and non-freshness of egg products and validation of molecules	(UHPLC–HRMS); untargeted	SIMCA, PCA, and OPLS-DA	[106]
Quality/Nutritional	Investigation of metabolite profile variations during industrial pasta processing for five different commercial products	GC–MS and LC–MS	ANOVA, PCA, and factor analysis	[74]
Nutritional	Metabolite profile and discrimination among the different germinated rice (black, red, and white)	^1^H NMR	PCA and PLS-DA	[100]
Nutritional	Investigation of metabolic changes following dietary intervention with soy isoflavones in healthy of premenopausal women	^1^H NMR and RP-HPLC–DAD	PCA and SIMCA-P	[107]

**Table 3:** Examples of metabolomic approach in food processing in foods.

Application in Food Processing	Detection Technology	Data Treatment	References
Variation in phytochemical profiles (carotenoids, flavonoids, glucosinolates, volatiles) of tomato, broccoli, and carrot purees modifying the processing order (between blending and heat treatment)	HPLC-PDA, GC–MS, ^1^H NMR, RP-LC-PDA-QTOF MS, and GC–MS for volatiles components	PCA and Student’s *t*-tests	[40]
Investigation of metabolomic profiling of chia, linseed, and sesame as processing-dependent biomarkers in cookies production	GC–MS	PCA and RF	[110]
Investigation of metabolite profile variations (phytos-terols, hydroxy fatty acids, tocopherols, and carotenoids) during industrial semolina pasta processing for five different commercial products	GC–MS and LC–MS	ANOVA, PCA	[74]
Investigation of different marinades in chicken breast fillets. Combination between pomegranate and lemon juice, probably due to the synergistic effect of organic acids (lemon juice) and polyphenols (pomegranate juice), provided the high decrease in *Pseudomonas* spp. bacteria	HPLC system	PCA	[120]
Investigation of changes in metabolite composition of marinated meat in soy sauce during processing as taste quality is directly related to primary and secondary metabolites	^1^H NMR	PCA, OPLS-DA, and ANOVA	[121]
Study on metabolomics of lettuce and the changes after storage of two cultivars with different susceptibility to browning. Tendency showed high amounts of phenolic compounds, fatty acids, and lysophospholipid with the storage time (day 5) and with the browning process	UPLC–ESI-QTOF-MS (untargeted)	PCA and HCA (unsupervised methods)	[122]
Investigation of the relationship between specific metabolites and the plant matrix with glucosinolate thermal degradation during food processing of *Brassica* vegetables. The interest is to minimize losses of glucosinolate during vegetable processing	HPLC-PDA-QTOF MS (untargeted)	PCA, HCA and RF	[123]
Optimization by applying metabolic profiling method to study the effect of typical domestic storage conditions for five red wines for a period of 24 months. Storage conditions had a major impact on the polar metabolite fingerprint, and the markers revealed included phenolic compounds, vitamins, and 4-amino-heptanedioic acid and its ethyl ester	UPLC–QTOF-MS (untargeted)	PCA, OPLS-DA, *t*-test, *U*-test, and *S*-plot	[124]
Investigation of distinctions in the phenolic profile of tomato products (crushed pulp, puree, and paste) and in tomato paste under three different treatments (cold, warm, and hot). Distinctions were possible to identify, especially in relation to flavonoids, phenylpropanoids, and lignans, as well as distinctions between the production location	UHPLC/Q-TOF	ANOVA, HCA, (unsupervised) and PLS-DA	[118]
Investigation of chemical profile changes resulting from thermal processing of black raspberries powder into a nectar beverage with a metabolomics approach. Degradation products of anthocyanins were identified along with other proposed phenolic degradants, while quercetin, phenolic acids, and ellagic acid were relatively stable to processing	UHPLC–QTOF-MS (Untargeted)	HCA	[125]
Investigation of metabolic changes during post-harvest of *Salvia miltiorrhiza* Bunge. The processing demonstrated great impacts on phenolic acids than on tanshinones, and enzymatic browning was the major influencing factor during post-harvest processing. The data showed that the reduction of the enzymatic browning could be achieved by controlling the moisture and steaming process	UHPLC–QTOF-MS	PCA (unsupervised), PLS-DA, and OPLS-DA	[126]
Investigation of metabolomics and proteomics to study the change mechanism of nonvolatile compounds during white tea processing. Decreased content of catechins, proanthocyanidins, thasins, and phenolic acids and increased content of free amino acids, theaflavins, and nucleotides are responsible for the sweet taste of tea. The drying process was found to promote the formation of white tea taste	UPLC–LTQ-Orbitrap-MS (untargeted)	PCA and ANOVA	[127]

## References

[1] Coulier L., Wopereis S., Rubingh C., Hendriks H., Radonjic´ M., Jellema R.H. (2009). Systems Biology. An Introduction to Key Concepts in Medicinal Chemistry.

[2] Benson D.A., Karsch-Mizrachi I., Lipman D.J., Ostell J., Rapp B.A., Wheeler D.L. (2002). GenBank. Nucleic Acids Res..

[3] Fanos V., Antonucci R., Barberini L., Atzori L. (2012). Urinary Metabolomics in Newborns and Infants. Adv. Clin. Chem..

[4] Park S.T., Kim J. (2016). Trends in next-generation sequencing and a new era for whole genome sequencing. Int. Neurourol. J..

[5] Lu X., Ji L., Chen J. (2014). Metabolomic Profiling of Neoplastic Lesions in Mice. Cell-Wide Metabolic Alterations Associated with Malignancy.

[6] Dettmer K., Aronov P.A., Hammock B.D. (2006). Mass spectrometry-based metabolomics. Mass Spectrom. Rev..

[7] Liu R., Yang Z. (2021). Single cell metabolomics using mass spectrometry: Techniques and data analysis. Anal. Chim. Acta.

[8] Dwivedi G.R., Sisodia B.S., Shikha, Gupta V.K., Pandey A. (2019). Secondary Metabolites: Metabolomics for Secondary Metabolites. New and Future Developments in Microbial Biotechnology and Bioengineering: Microbial Secondary Metabolites Biochesmistry and Applications.

[9] Hall R.D. (2006). Plant metabolomics: From holistic hope, to hype, to hot topic. New Phytol..

[10] De Castro M.D.L., Priego-Capote F. (2018). The analytical process to search for metabolomics biomarkers. J. Pharm. Biomed. Anal..

[11] Jiang L., Howlett K., Patterson K., Wang B. (2020). Introduction of a new method for two-dimensional NMR quantitative analysis in metabolomics studies. Anal. Biochem..

[12] Rocchetti G., O’Callaghan T.F. (2021). Application of metabolomics to assess milk quality and traceability. Curr. Opin. Food Sci..

[13] Castro-Puyana M., Herrero M. (2013). Metabolomics approaches based on mass spectrometry for food safety, quality and traceability. Trends Anal. Chem..

[14] Lu Y., Chen C. (2017). Metabolomics: Bridging Chemistry and Biology in Drug Discovery and Development. Curr. Pharmacol. Rep..

[15] Campillo J.A., Sevilla A., González-Fernández C., Bellas J., Bernal C., Cánovas M., Albentosa M. (2019). Metabolomic responses of mussel Mytilus galloprovincialis to fluoranthene exposure under different nutritive conditions. Mar. Environ. Res..

[16] Wu H., Wang W.X. (2010). NMR-based metabolomic studies on the toxicological effects of cadmium and copper on green mussels Perna viridis. Aquat. Toxicol..

[17] Zhang L., Liu X., You L., Zhou D., Wang Q., Li F., Cong M., Li L., Zhao J., Liu D. (2011). Benzo(a)pyrene-induced metabolic responses in Manila clam Ruditapes philippinarum by proton nuclear magnetic resonance (^1^H NMR) based metabolomics. Environ. Toxicol. Pharmacol..

[18] Murithi J.M., Owen E.S., Istvan E.S., Lee M.C.S., Ottilie S., Chibale K., Goldberg D.E., Winzeler E.A., Llinás M., Fidock D.A. (2020). Combining Stage Specificity and Metabolomic Profiling to Advance Antimalarial Drug Discovery. Cell Chem. Biol..

[19] Klont F., Kremer D., Gomes Neto A.W., Berger S.P., Touw D.J., Hak E., Bonner R., Bakker S.J.L., Hopfgartner G. (2021). Metabolomics data complemented drug use information in epidemiological databases: Pilot study of potential kidney donors. J. Clin. Epidemiol..

[20] Cramer G.R., Urano K., Delrot S., Pezzotti M., Shinozaki K. (2011). Effects of abiotic stress on plants: A systems biology perspective. BMC Plant Biol..

[21] Almuhayawi M.S., Hassan A.H.A., Al S.K. (2021). Influence of elevated CO 2 on nutritive value and health-promoting prospective of three genotypes of Alfalfa sprouts (*Medicago Sativa*). Food Chem..

[22] Cozzolino D. (2015). Foodomics and infrared spectroscopy: From compounds to functionality. Curr. Opin. Food Sci..

[23] Jacobs D.M., van den Berg M.A., Hall R.D. (2021). Towards superior plant-based foods using metabolomics. Curr. Opin. Biotechnol..

[24] Kim S., Kim J., Yun E.J., Kim K.H. (2016). Food metabolomics: From farm to human. Curr. Opin. Biotechnol..

[25] Pavagadhi S., Swarup S. (2020). Metabolomics for evaluating flavor-associated metabolites in plant-based products. Metabolites.

[26] Wu B., Wei F., Xu S., Xie Y., Lv X., Chen H., Huang F. (2021). Mass spectrometry-based lipidomics as a powerful platform in foodomics research. Trends Food Sci. Technol..

[27] Cifuentes A. (2009). Food analysis and foodomics. J. Chromatogr. A.

[28] Skov T., Honoré A.H., Jensen H.M., Næs T., Engelsen S.B. (2014). Chemometrics in foodomics: Handling data structures from multiple analytical platforms. Trends Anal. Chem..

[29] Steuer A.E., Brockbals L., Kraemer T. (2019). Metabolomic strategies in biomarker research-new approach for indirect identification of drug consumption and sample manipulation in clinical and forensic toxicology?. Front. Chem..

[30] Sanchez S., Demain A.L. (2008). Metabolic regulation and overproduction of primary metabolites. Microb. Biotechnol..

[31] Canarini A., Kaiser C., Merchant A., Richter A., Wanek W. (2019). Root exudation of primary metabolites: Mechanisms and their roles in plant responses to environmental stimuli. Front. Plant Sci..

[32] Campos M.R.S. (2019). Bioactive Compounds: Health Benefits and Potential Applications.

[33] Aharoni A., Galili G. (2011). Metabolic engineering of the plant primary-secondary metabolism interface. Curr. Opin. Biotechnol..

[34] Tyc O., Song C., Dickschat J.S., Vos M., Garbeva P. (2017). The Ecological Role of Volatile and Soluble Secondary Metabolites Produced by Soil Bacteria. Trends Microbiol..

[35] Bills G.F., Gloer J.B. (2017). Biologically active secondary metabolites from the fungi. The Fungal Kingdom.

[36] Rastegari A.A., Yadav A.N., Yadav N., Gupta V.K., Pandey A. (2019). Genetic Manipulation of Secondary Metabolites Producers. New and Future Developments in Microbial Biotechnology and Bioengineering: Microbial Secondary Metabolites Biochesmistry and Applications.

[37] Gokulan K., Khare S., Cerniglia C. (2014). Production of Secondary Metabolites of Bacteria. Encyclopedia of Food Microbiology.

[38] Kumar A., Naraian R., Gupta V.K., Pandey A. (2019). Producers of Bioactive Compounds. New and Future Developments in Microbial Biotechnology and Bioengineering: Microbial Secondary Metabolites Biochesmistry and Applications.

[39] Toffano R.B.D., Hillesheim E., Mathias M.G., Coelho-Landell C.A., Salomão R.G., Almada M.O.R.V., Camarneiro J.M., Barros T.T., Camelo-Junior J.S., Rezzi S. (2018). Validation of the brazilian healthy eating index-revised using biomarkers in children and adolescents. Nutrients.

[40] Lopez-Sanchez P., De Vos R.C.H., Jonker H.H., Mumm R., Hall R.D., Bialek L., Leenman R., Strassburg K., Vreeken R., Hankemeier T. (2015). Comprehensive metabolomics to evaluate the impact of industrial processing on the phytochemical composition of vegetable purees. Food Chem..

[41] Capuano E., Boerrigter-Eenling R., van der Veer G., van Ruth S.M. (2013). Analytical authentication of organic products: An overview of markers. J. Sci. Food Agric..

[42] Li M., Wang X., Han L., Jia L., Liu E., Li Z., Yu H., Wang Y., Gao X., Yang W. (2020). Integration of multicomponent characterization, untargeted metabolomics and mass spectrometry imaging to unveil the holistic chemical transformations and key markers associated with wine steaming of Ligustri Lucidi Fructus. J. Chromatogr. A.

[43] Amargianitaki M., Spyros A. (2017). NMR-based metabolomics in wine quality control and authentication. Chem. Biol. Technol. Agric..

[44] Giacometti J., Tomljanović A.B., Josić D. (2013). Application of proteomics and metabolomics for investigation of food toxins. Food Res. Int..

[45] Oyedeji A.B., Green E., Adebiyi J.A., Ogundele O.M., Gbashi S., Adefisoye M.A., Oyeyinka S.A., Adebo O.A. (2021). Metabolomic Approaches for the Determination of Metabolites from Pathogenic Microorganisms: A Review. Food Res. Int..

[46] Rawat J.M., Bhandari A., Raturi M., Rawat B., Gupta V.K., Pandey A. (2019). Agrobacterium rhizogenes Mediated Hairy Root Cultures: A Promising Approach for Production of Useful Metabolites. New and Future Developments in Microbial Biotechnology and Bioengineering: Microbial Secondary Metabolites Biochesmistry and Applications.

[47] Keller N.P., Turner G., Bennett J.W. (2005). Fungal secondary metabolism—From biochemistry to genomics. Nat. Rev. Microbiol..

[48] Zhong J.-J., Xiao J.-H., Zhong J.-J., Bai F.-W., Zhang W. (2009). Secondary Metabolites from Higher Fungi: Discovery, Bioactivity, and Bioproduction. Biotechnology in China I. From Bioreaction to Bioseparation and Bioremediation.

[49] Andryukov B., Mikhailov V., Besednova N. (2019). The biotechnological potential of secondary metabolites from marine bacteria. J. Mar. Sci. Eng..

[50] Mohan C.D., Rangappa S., Nayak S.C., Jadimurthy R., Wang L., Sethi G., Garg M., Rangappa K.S. (2021). Bacteria as a treasure house of secondary metabolites with anticancer potential. Semin. Cancer Biol..

[51] Hamacher M., Malisch C.S., Reinsch T., Taube F., Loges R. (2021). Evaluation of yield formation and nutritive value of forage legumes and herbs with potential for diverse grasslands due to their concentration in plant specialized metabolites. Eur. J. Agron..

[52] Liu Z., Sun J., Teng Z., Luo Y., Yu L., Simko I., Chen P. (2021). Identification of marker compounds for predicting browning of fresh-cut lettuce using untargeted UHPLC-HRMS metabolomics. Postharvest Biol. Technol..

[53] Pereira G.E., Padhi E.M.T., Sudarshana M.R., Fialho F.B., Medina-Plaza C., Girardello R.C., Tseng D., Bruce R.C., Erdmann J.N., Slupsky C.M. (2021). Impact of grapevine red blotch disease on primary and secondary metabolites in ‘Cabernet Sauvignon’ grape tissues. Food Chem..

[54] Zhan J., Yu X.J., Zhong Y.Y., Zhang Z.T., Cui X.M., Peng J.F., Feng R., Liu X.T., Zhu Y. (2012). Generic and rapid determination of veterinary drug residues and other contaminants in raw milk by ultra performance liquid chromatography-tandem mass spectrometry. J. Chromatogr. B Anal. Technol. Biomed. Life Sci..

[55] Hanson J.R. (2003). Natural Products: The Secondary Metabolites. Tutorial Chemistry Texts.

[56] Bajkacz S., Kycia-Słocka E., Pico Y. (2020). Liquid chromatography in food analysis. Chemical Analysis of Food—Techniques and Applications.

[57] Cubero-Leon E., Peñalver R., Maquet A. (2014). Review on metabolomics for food authentication. Food Res. Int..

[58] Choi M.Y., Choi W., Park J.H., Lim J., Kwon S.W. (2010). Determination of coffee origins by integrated metabolomic approach of combining multiple analytical data. Food Chem..

[59] Cevallos-cevallos J.M., Etxeberria E., Danyluk M.D., Rodrick G.E. (2009). Metabolomic analysis in food science: A review. Trends Food Sci. Technol..

[60] Oms-Oliu G., Odriozola-Serrano I., Martín-Belloso O. (2013). Metabolomics for assessing safety and quality of plant-derived food. Food Res. Int. J..

[61] Mamat S.F., Azizan K.A., Baharum S.N., Noor N.M., Aizat W.M. (2020). GC-MS and LC-MS analyses reveal the distribution of primary and secondary metabolites in mangosteen (*Garcinia mangostana* Linn.) fruit during ripening. Sci. Hortic..

[62] Sørensen K.M., Khakimov B., Engelsen S.B. (2016). The use of rapid spectroscopic screening methods to detect adulteration of food raw materials and ingredients. Curr. Opin. Food Sci..

[63] Mairinger T., Causon T.J., Hann S. (2018). The potential of ion mobility–mass spectrometry for non-targeted metabolomics. Curr. Opin. Chem. Biol..

[64] Lebanov L., Ghiasvand A., Paull B. (2021). Data handling and data analysis in metabolomic studies of essential oils using GC-MS. J. Chromatogr. A.

[65] Zhang J., Hu Q., Yu Q., Chen Y., Zhao Y., Qie M. (2020). Metabolomics Analysis in Food Authentication. Comprehensive Foodomics.

[66] Vu Dang H., Marini F. (2019). Editorial: Chemometrics-based spectroscopy for pharmaceutical and biomedical analysis. Front. Chem..

[67] Oliveri P., Malegori C., Casale M., Pico Y. (2020). Chemometrics: Multivariate analysis of chemical data. Chemical Analysis of Food—Techniques and Applications.

[68] Sparkman O.D., Penton Z.E., Kitson F.G. (2011). Gas Chromatography and Mass Spectrometry: A Practical Guide.

[69] McNair H.M., Miller J.M., Snow N.H. (2019). Basic Gas Chromatography.

[70] Engewald W., Dettmer-Wilde K., Dettmer-Wilde K., Engewald W. (2014). Practical Gas Chromatography: A Comprehensive Reference.

[71] Jennings W., Mittlefehldt E., Stremple P. (1997). Analytical Gas Chromatography.

[72] Mihailova A., Kelly S.D., Chevallier O.P., Elliott C.T., Maestroni B.M., Cannavan A. (2021). High-resolution mass spectrometry-based metabolomics for the discrimination between organic and conventional crops: A review. Trends Food Sci. Technol..

[73] Tan K., Ipcho S.V.S., Trengove R.D., Oliver R.P., Solomon P.S. (2009). Challenges for molecular plant pathology over the next ten years: Assessing the impact of transcriptomics, proteomics and metabolomics on fungal phytopathology. Mol. Plant Pathol..

[74] Beleggia R., Platani C., Papa R., Di Chio A., Barros E., Mashaba C., Wirth J., Fammartino A., Sautter C., Conner S. (2011). Metabolomics and food processing: From semolina to pasta. J. Agric. Food Chem..

[75] Palamareva M.D., Worsfold P., Townshend A., Poole C. (2005). Liquid Chromatography: Overview. Encyclopedia of Analytical Science.

[76] Swartz M., Worsfold P., Townshend A., Poole C. (2005). Liquid chromatography: Mobile phase selection. Encyclopedia of Analytical Science.

[77] Meyer V.R. (2010). Practical High-Performance Liquid Chromatography.

[78] Cubero-Leon E., De Rudder O., Maquet A. (2018). Metabolomics for organic food authentication: Results from a long-term field study in carrots. Food Chem..

[79] do Lago C.L., Daniel D., Lopes F.S., Cieslarová Z., Yolanda P. (2020). Electrophoresis. Chemical Analysis of Food—Techniques and Applications.

[80] Stringer R., Worsfold P., Townshend A., Poole C. (2005). Electrophoresis: Overview. Encyclopedia of Analytical Science.

[81] Bowser M., Worsfold P., Townshend A., Poole C. (2005). Capillary Electrophoresis: Overview. Encyclopedia of Analytical Science.

[82] Snyder L.R., Kirkland J.J., Dolan J.W. (2010). Introduction to Modern Liquid Chromatography. J. Am. Soc. Mass Spectrom..

[83] Ibáñez C., Simó C., García-Cañas V., Cifuentes A., Castro-Puyana M. (2013). Metabolomics, peptidomics and proteomics applications of capillary electrophoresis-mass spectrometry in Foodomics: A review. Anal. Chim. Acta.

[84] García-Villalba R., León C., Dinelli G., Segura-Carretero A., Fernández-Gutiérrez A., Garcia-Cañas V., Cifuentes A. (2008). Comparative metabolomic study of transgenic versus conventional soybean using capillary electrophoresis-time-of-flight mass spectrometry. J. Chromatogr. A.

[85] Levandi T., Leon C., Kaljurand M., Garcia-Cañas V., Cifuentes A. (2008). Capillary electrophoresis time-of-flight mass spectrometry for comparative metabolomics of transgenic versus conventional maize. Anal. Chem..

[86] Zhang X., Quinn K., Cruickshank-Quinn C., Reisdorph R., Reisdorph N. (2018). The application of ion mobility mass spectrometry to metabolomics. Curr. Opin. Chem. Biol..

[87] Feuerstein M.L., Kurulugama R.T., Hann S., Causon T. (2021). Novel acquisition strategies for metabolomics using drift tube ion mobility-quadrupole resolved all ions time-of-flight mass spectrometry (IM-QRAI-TOFMS). Anal. Chim. Acta.

[88] Wishart D.S. (2019). Perspectives in Magnetic Resonance NMR metabolomics: A look ahead. J. Magn. Reson..

[89] Hatzakis E. (2019). Nuclear Magnetic Resonance (NMR) Spectroscopy in Food Science: A Comprehensive Review. Compr. Rev. Food Sci. Food Saf..

[90] Janovick J., Spyros A., Dais P., Hatzakis E., Pico Y. (2020). Nuclear magnetic resonance. Chemical Analysis of Food—Techniques and Applications.

[91] Emwas A., Roy R., Mckay R.T., Tenori L., Saccenti E., Gowda G.A.N., Raftery D., Alahmari F., Jaremko L., Jaremko M. (2019). NMR Spectroscopy for Metabolomics Research. Metabolites.

[92] Laghi L., Picone G., Capozzi F. (2014). Nuclear magnetic resonance for foodomics beyond food analysis. Trends Anal. Chem..

[93] Kaklamanos G., Aprea E., Theodoridis G., Pico Y. (2020). Mass spectrometry: Principles and instrumentation. Chemical Analysis of Food—Techniques and Applications.

[94] Dunn W.B. (2008). Current trends and future requirements for the mass spectrometric investigation of microbial, mammalian and plant metabolomes. Phys. Biol..

[95] Lee D.K., Yoon M.H., Kang Y.P., Yu J., Park J.H., Lee J., Kwon S.W. (2013). Comparison of primary and secondary metabolites for suitability to discriminate the origins of Schisandra chinensis by GC/MS and LC/MS. Food Chem..

[96] Okada T., Mochamad Afendi F., Altaf-Ul-Amin M., Takahashi H., Nakamura K., Kanaya S. (2010). Metabolomics of Medicinal Plants: The Importance of Multivariate Analysis of Analytical Chemistry Data. Curr. Comput. Aided Drug Des..

[97] Society M. (2014). Metabolomic Database. http://metabolomicssociety.org/resources/metabolomics-databases.

[98] Reisdorph N., Reisdorph R., Quinn K., Doenges K. (2019). Metabolomics Mass Spectrometry Data Processing: Applications in Food Analysis. Comprehensive Foodomics.

[99] Reid L.M., O’Donnell C.P., Downey G. (2004). Potential of SPME-GC and Chemometrics to Detect Adulteration of Soft Fruit Purées. J. Agric. Food Chem..

[100] Pramai P., Abdul Hamid N.A., Mediani A., Maulidiani M., Abas F., Jiamyangyuen S. (2018). Metabolite profiling, antioxidant, and α-glucosidase inhibitory activities of germinated rice: Nuclear-magnetic-resonance-based metabolomics study. J. Food Drug Anal..

[101] Wei F., Wu B. (2020). Use of Lipidomics for Food Quality Assurance and Authentication. Comprehensive Foodomics.

[102] European Parliament, Council of the European Union (2011). Regulation (EU) No 1169/2011 of the European Parliament and of the Council of 25 October 2011 on the provision of food information to consumers, amending Regulations (EC) No 1924/2006 and (EC) No 1925/2006 of the European Parliament and of the Council. Off. J. Eur. Union.

[103] European Comission (2021). The EU Food Fraud Network. Food Safety.

[104] European Comission (2021). Quality Schemes Explained.

[105] FAO/WHO (2021). Nutrition and Labelling. Codex Alimentarius International Food Standards.

[106] Cavanna D., Catellani D., Dall’asta C., Suman M. (2018). Egg product freshness evaluation: A metabolomic approach. J. Mass Spectrom..

[107] Solanky K.S., Bailey N.J.C., Beckwith-Hall B.M., Davis A., Bingham S., Holmes E., Nicholson J.K., Cassidy A. (2003). Application of biofluid 1H nuclear magnetic resonance-based metabonomic techniques for the analysis of the biochemical effects of dietary isoflavones on human plasma profile. Anal. Biochem..

[108] Wang H., Hua J., Yu Q., Li J., Wang J., Deng Y., Yuan H. (2021). Widely targeted metabolomic analysis reveals dynamic changes in non-volatile and volatile metabolites during green tea processing. Food Chem..

[109] Ikeda T., Kanaya S., Yonetani T., Kobayashi A., Fukusaki E. (2007). Prediction of Japanese green tea ranking by fourier transform near-infrared reflectance spectroscopy. J. Agric. Food Chem..

[110] Erban A., Fehrle I., Martinez-Seidel F., Brigante F., Más A.L., Baroni V., Wunderlin D., Kopka J. (2019). Discovery of food identity markers by metabolomics and machine learning technology. Sci. Rep..

[111] Rešetar D., Pavelić S.K., Josić D. (2015). Foodomics for investigations of food toxins. Curr. Opin. Food Sci..

[112] Giacometti J., Josic D. (2013). Foodomics in microbial safety. Trends Anal. Chem..

[113] Ulaszewska M.M., Weinert C.H., Trimigno A., Portmann R., Andres Lacueva C., Badertscher R., Brennan L., Brunius C., Bub A., Capozzi F. (2019). Nutrimetabolomics: An Integrative Action for Metabolomic Analyses in Human Nutritional Studies. Mol. Nutr. Food Res..

[114] Wishart D.S. (2008). Metabolomics: Applications to food science and nutrition research. Trends Food Sci. Technol..

[115] Gibney M.J., Walsh M., Brennan L., Roche H.M., German B., Van Ommen B. (2005). Metabolomics in human nutrition: Opportunities and challenges. Am. J. Clin. Nutr..

[116] Alfaro A.C., Young T. (2018). Showcasing metabolomic applications in aquaculture: A review. Rev. Aquac..

[117] Utpott M., Rodrigues E., de Rios A.O., Mercali G.D., Flôres S.H. (2022). Metabolomics: An analytical technique for food processing evaluation. Food Chem..

[118] Lucini L., Rocchetti G., Kane D., Trevisan M. (2017). Phenolic fingerprint allows discriminating processed tomato products and tracing different processing sites. Food Control.

[119] Rubert J., Zachariasova M., Hajslova J. (2015). Advances in high-resolution mass spectrometry based on metabolomics studies for food—A review. Food Addit. Contam. Part A Chem. Anal. Control. Expo Risk Assess..

[120] Lytou A.E., Panagou E.Z., Nychas G.J.E. (2017). Effect of different marinating conditions on the evolution of spoilage microbiota and metabolomic profile of chicken breast fillets. Food Microbiol..

[121] Yang Y., Ye Y., Pan D., Sun Y., Wang Y., Cao J. (2018). Metabonomics profiling of marinated meat in soy sauce during processing. J. Sci. Food Agric..

[122] Garcia C.J., García-Villalba R., Garrido Y., Gil M.I., Tomás-Barberán F.A. (2016). Untargeted metabolomics approach using UPLC-ESI-QTOF-MS to explore the metabolome of fresh-cut iceberg lettuce. Metabolomics.

[123] Hennig K., De Vos R.C.H., Maliepaard C., Dekker M., Verkerk R., Bonnema G. (2014). A metabolomics approach to identify factors influencing glucosinolate thermal degradation rates in *Brassica* vegetables. Food Chem..

[124] Arapitsas P., Della Corte A., Gika H., Narduzzi L., Mattivi F., Theodoridis G. (2016). Studying the effect of storage conditions on the metabolite content of red wine using HILIC LC-MS based metabolomics. Food Chem..

[125] Teegarden M.D., Schwartz S.J., Cooperstone J.L. (2019). Profiling the impact of thermal processing on black raspberry phytochemicals using untargeted metabolomics. Food Chem..

[126] Qiu S., Tu Y., Huang D., Dong Z., Huang M., Cheng J., Tan J., Chen W., Sun L., Chen W. (2021). Selection of appropriate post-harvest processing methods based on the metabolomics analysis of *Salvia miltiorrhiza* Bunge. Food Res. Int..

[127] Chen Q., Shi J., Mu B., Chen Z., Dai W., Lin Z. (2020). Metabolomics combined with proteomics provides a novel interpretation of the changes in nonvolatile compounds during white tea processing. Food Chem..

